# Supplementary material for: Neural representational geometries reflect behavioral differences in monkeys and recurrent neural networks
Source: Nat Commun. 2024 Aug 1;15:6479. doi: 10.1038/s41467-024-50503-w (PMC11294567; doi:10.1038/s41467-024-50503-w)
Supplement: Supplementary file 1 — Supplementary Information [file 41467_2024_50503_MOESM1_ESM.pdf]

Supplementary Figures

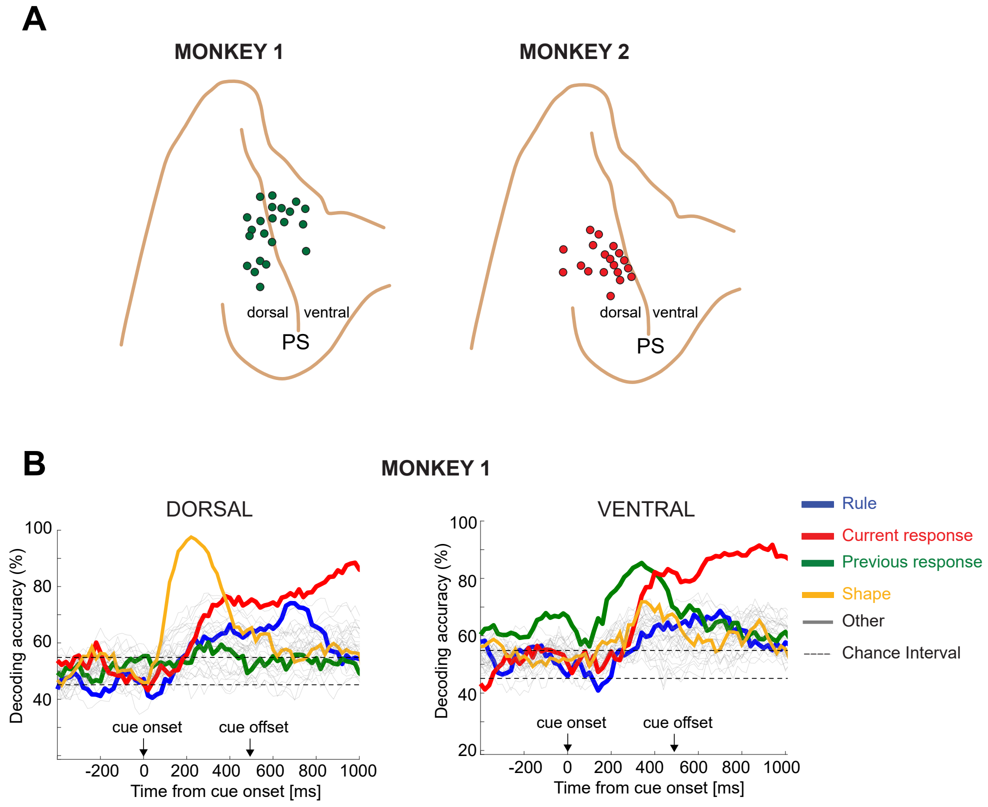

Fig. S1: **Recording sites for Monkey 1 and Monkey 2 in the dorsolateral prefrontal cortex (PFdl). A)** Recording sites in PFdl for the two monkeys. **B)** Decoding accuracies of all dichotomies for Monkey 1 after splitting neurons in dorsal (106 neurons) and ventral (99 neurons) recordings with respect to the principal sulcus. PS: principal sulcus.

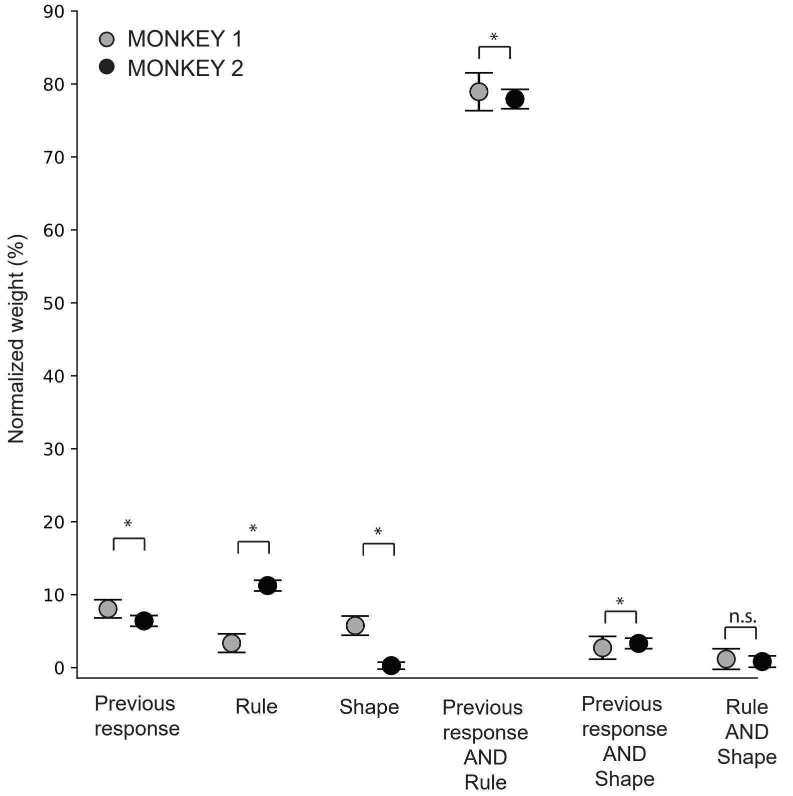

Fig. S2: **Multi-Linear regression analysis results**.Mean of the distribution of the weights of 100 multi-linear regression models. The reaction time is predicted on a single trial using three factors: previous response, rule, and shape along with the interaction terms. The interaction term of the previous response with the rule is the strongest factor in both monkeys since the combination of these two variables is essential to elaborate the correct response. The error bars are the 2 standard deviations of weights across 100 models. n.s.: not significant; * Mann-Whitney U test: $p<0.05$.

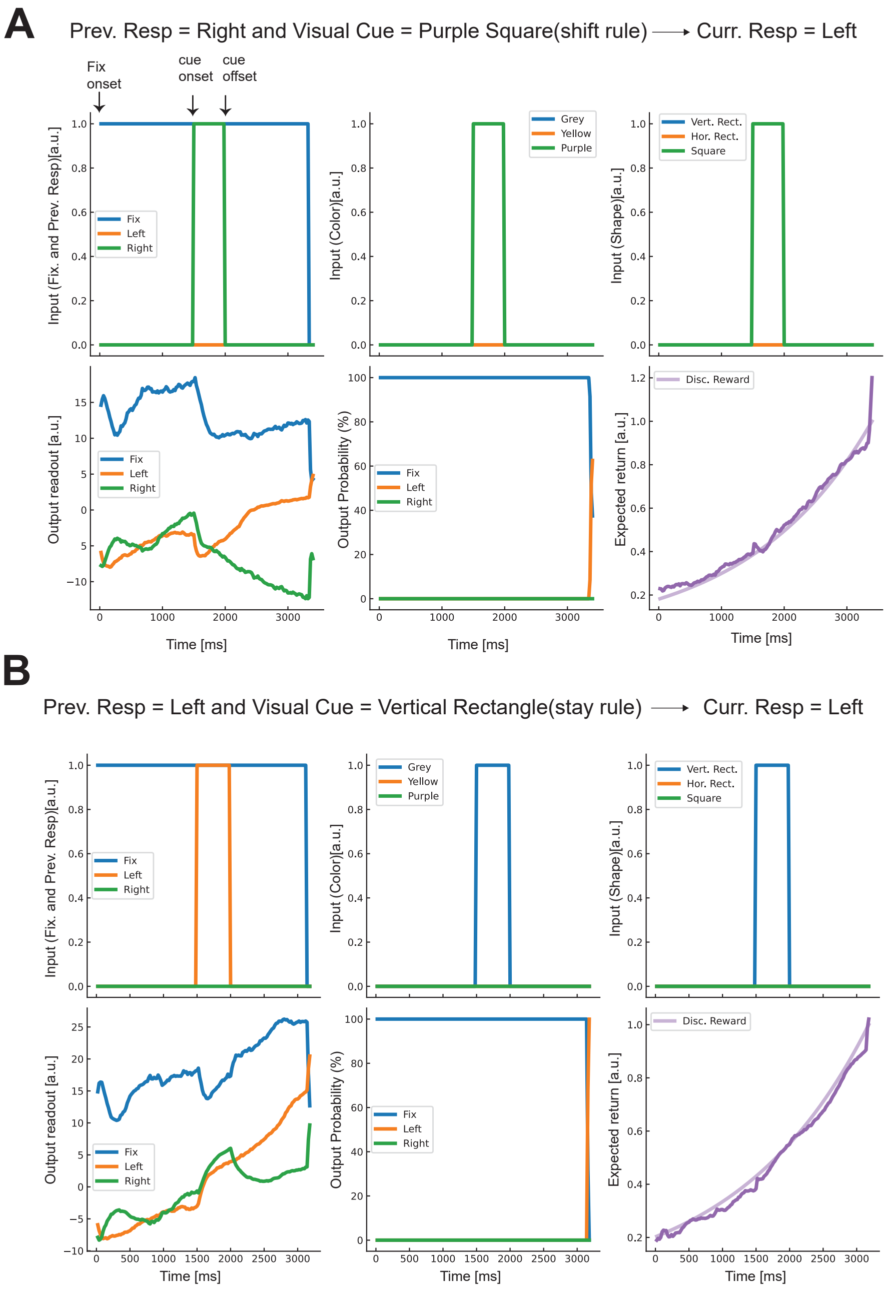

Fig. S3: **Example of two trials solved by an RNN. A)**. Example of a shift trial with the purple square visual cue and previous response right. The correct current response is left. The top row illustrates the three inputs to the network during the trial. Top-left: The fixation input (blue line) is constantly active before the go cue onset, while the previous response (green line) is given during the visual cue presentation for $500ms$. This is a simplified version of the real task where the animal is required to retrieve the previous response from the previous trial. Top-middle: color input (purple) of the visual cue that, in this example, is the purple square. The purple signal is presented during the cue presentation, and it lasted for $500ms$. Top-right: the shape input (square) of the visual cue that in this example is the purple square. The bottom row illustrates the different output readouts from the network during the trial. Bottom-left: example of the policy output signal during time. The fixation output signal is high while the fixation input is on (blue line). After the presentation of the visual cue, the left response signal (orange line) starts to increase over the right signal (green line) and the fixation signal. Bottom-middle: output probability computed from a softmax of the policy output signals at each time step during the trial. Bottom-right: Expected return is defined as the value function output along the trial: it decays exponentially backward in time because of the temporal discounting imposed during training. **B)** Example of a stay trial. This is the same as in A, with the left previous response, along with the vertical grey rectangle visual cue, requiring left as the current correct response.

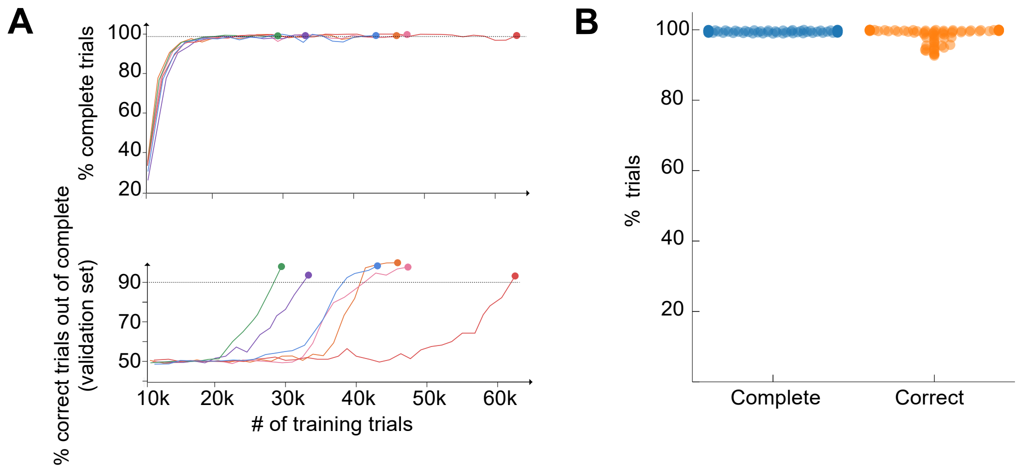

Fig. S4: **Percentage of complete and correct trials over complete trials of RNNs during training**. **A)** Top: Percentage of complete trials of six example RNNs as a function of the number of training trials. The horizontal dashed line indicates the threshold of 99% of complete trials where we stopped the training. Due to the heterogeneity, some networks converged earlier than others, potentially due to differences in initial random conditions and random pattern presentation of trial types during training. The percentage is calculated after a fixed number of time steps over a batch of 10000 validation trials (see Methods). Bottom: Percentage of correct trials out of the complete trials shown above for the same six RNNs. The horizontal dashed line indicates the threshold of 90% of correct trials where we stopped the training. The performance is assessed on a validation set not previously used for training. The high level of idiosyncrasy is evident among RNNs converging to the threshold at different amounts of training trials. **B)** Percentage of complete and correct of the complete trials for each of the 80 RNNs after training. All the RNNs satisfy the convergence criterion, i.e., at least 99% of complete trials (left) and at least 90% of correct trials out of the complete trials (right). The percentage is calculated over a batch of 10000 testing trials for each network.

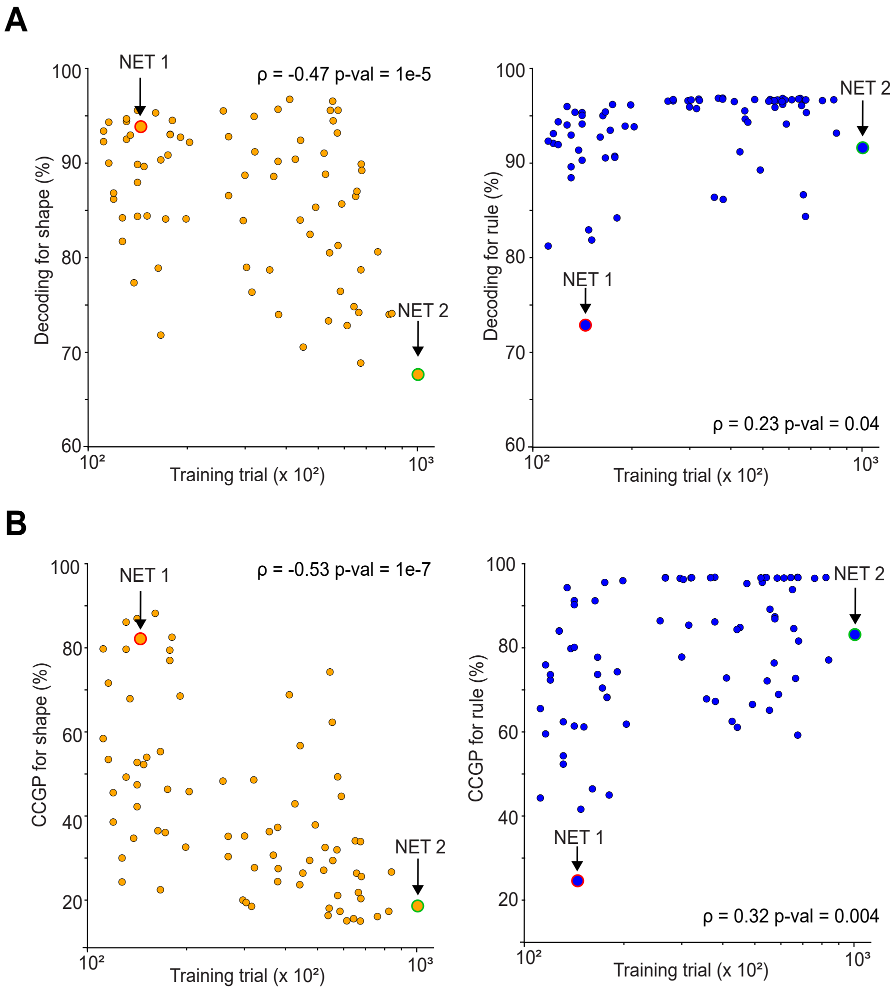

Fig. S5: **Decoding performance and CCGP for shape and rule during the cue presentation versus the number of training trials**. **A)** Left: Decoding accuracy of the shape during the 500ms of cue presentation as a function of the number of training trials required to reach the 90% of performance threshold. The higher the amount of training, the lower the decoding accuracy for the shape (Pearson correlation, ρ=-0.47, p-value=1e^{-}5). We enhanced the two example networks, NET 1 and NET 2, which resemble the monkeys' shape and rule representations (see Figure 8). Right: Decoding accuracy of the rule as a function of the training amount. The higher the training amount, the higher the decoding accuracy of the rule (Pearson correlation, ρ=0.23, p-value=0.04). **B)** Left: CCGP for the shape as a function of the amount of training. As for the decoding accuracy, the higher the amount of training, the lower the CCGP (Pearson correlation, ρ=-0.53, p-value=1e^{-}7). Right: CCGP for the rule as a function of the amount of training. The higher the amount of training, the higher the CCGP for the rule (Pearson correlation, ρ=0.32, p-value=0.004).

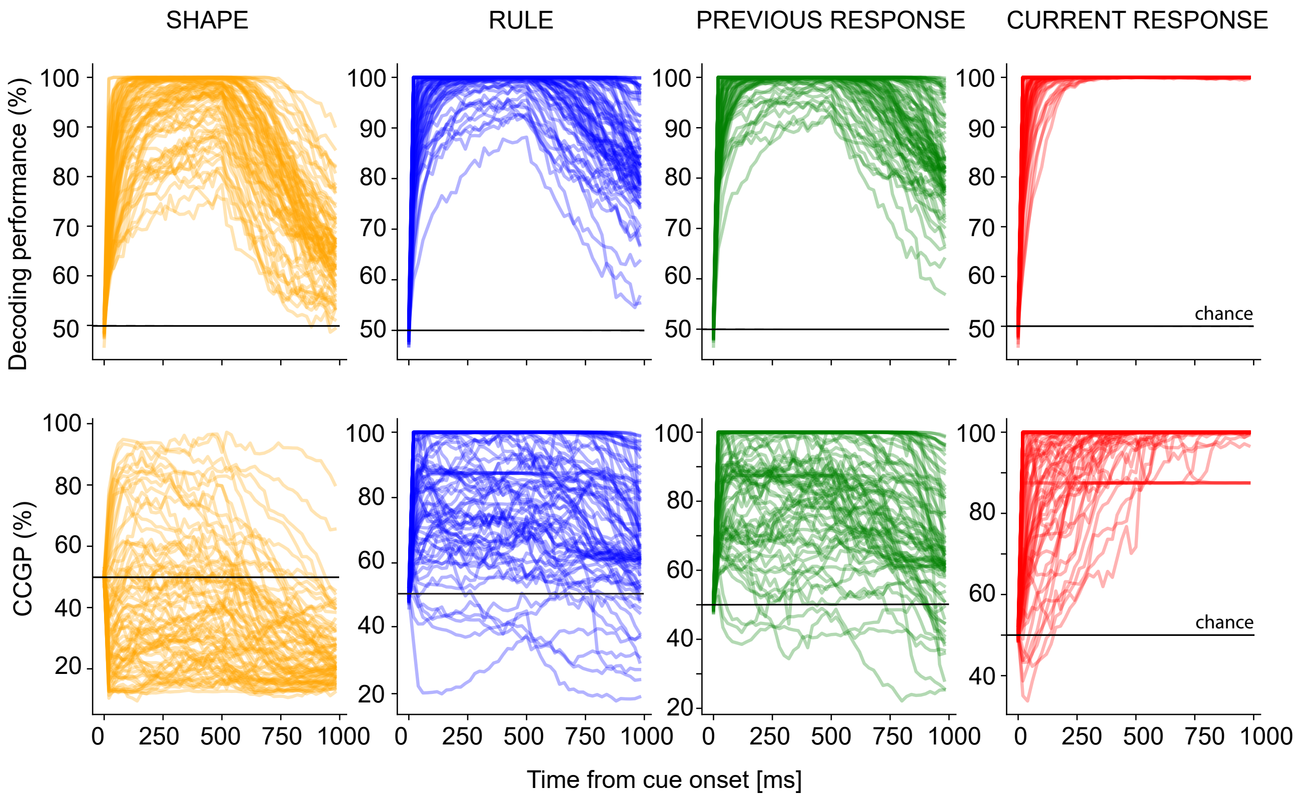

Fig. S6: **Decoding performance and CCGP of the four main task variables along time in all the trained RNNs**. Top-row: Decoding performance for the shape (orange), rule (blue), previous (green), and current (red) response, from the cue onset. The cue offset is at $500ms$ from cue onset, with the first $500ms$ of the delay. Bottom-row: CCGP for the shape (orange), rule (blue), previous (green), and current (red) response, along time from the cue onset.

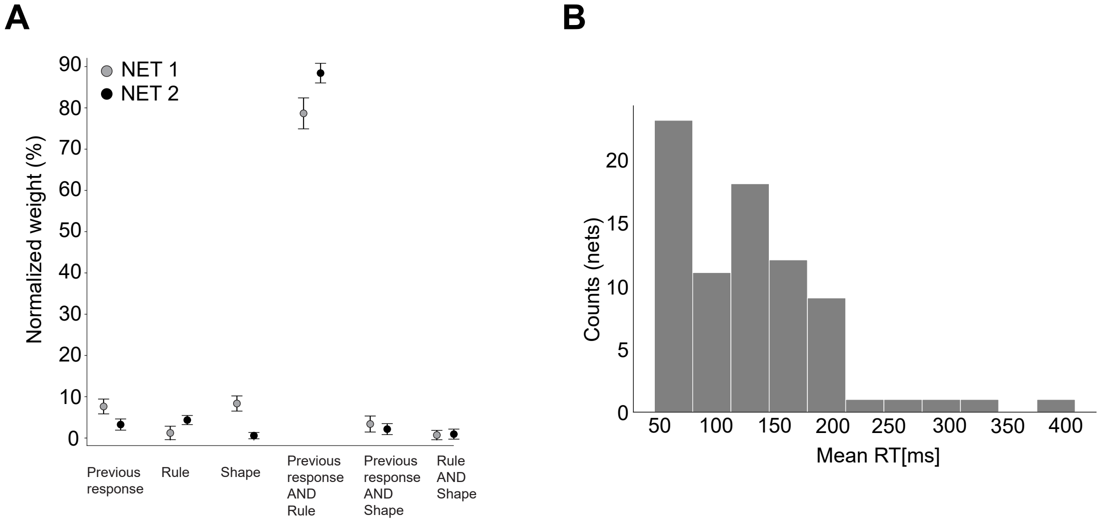

Fig. S7: **Average of multi-linear regression weights and distribution of reaction times across all the RNNs**. **A)** Mean of the distribution of the weights of 100 multi-linear regression models. The reaction time is predicted on a single trial using three factors: previous response, rule, and shape along with the interaction terms. The interaction term of the previous response with the rule is the strongest factor in both networks since the combination of these two variables is essential to elaborate the correct response. The error bars are the 2 standard deviations of weights across 100 models. **B)** Distribution of mean reaction times (RTs) for all the trained recurrent neural networks (RNNs). The mean of the distribution of RTs for NET 2 is smaller than the mean RTs in NET 1. This is due to the longer amount of training trials that allowed NET 2 to develop a better policy.

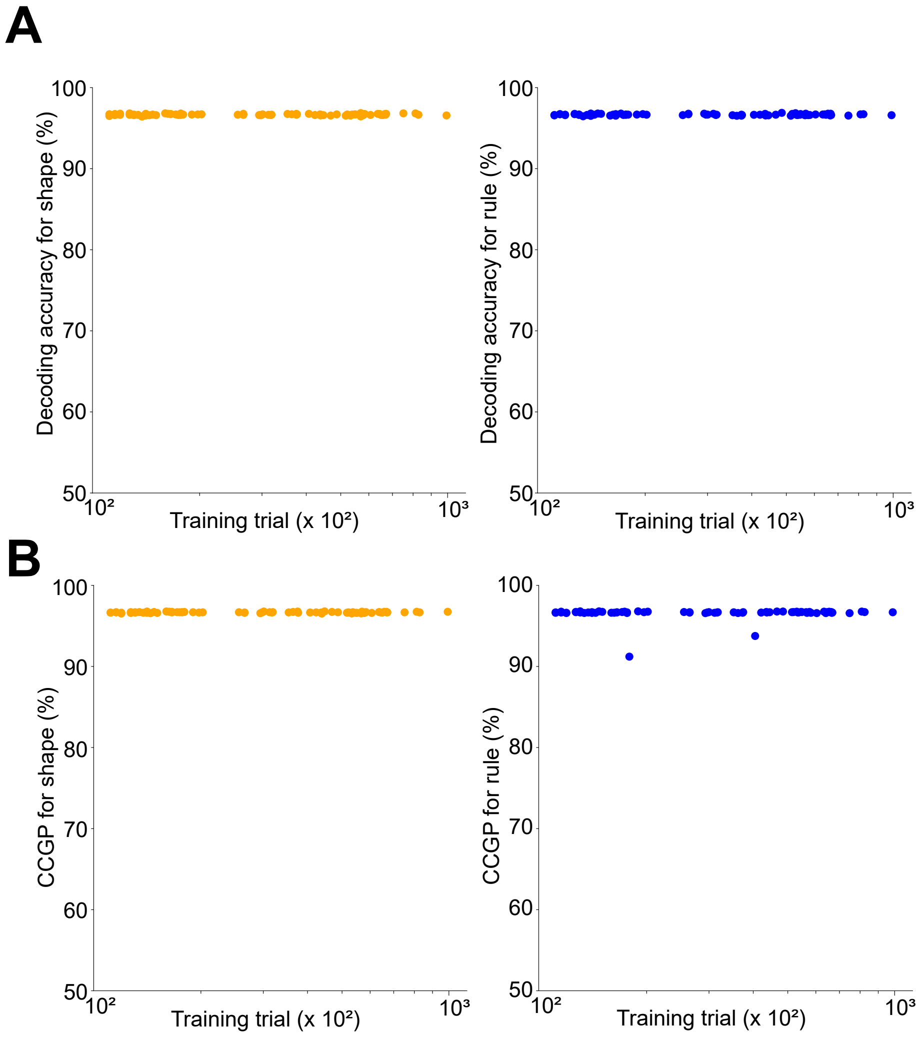

Fig. S8: **Decoding accuracy and CCGP for shape and rule in the input expansion layer of each RNN during the cue presentation as a function of the amount of training trials. A)** Decoding accuracy for shape (left) and rule (right) representation in the expansion input layer when the RNNs have reached the performance threshold. Each point is a different RNN. The accuracy is very high for each of the RNNs without any significant difference. The x-axis is the learning stage of each RNN defined as the amount of training trials required to reach the performance threshold. **B)** Same as in A but for the CCGP. In the expansion layer with random projection, the geometry still supports a high CCGP for both shape and rule across all the RNNs with no significant differences.

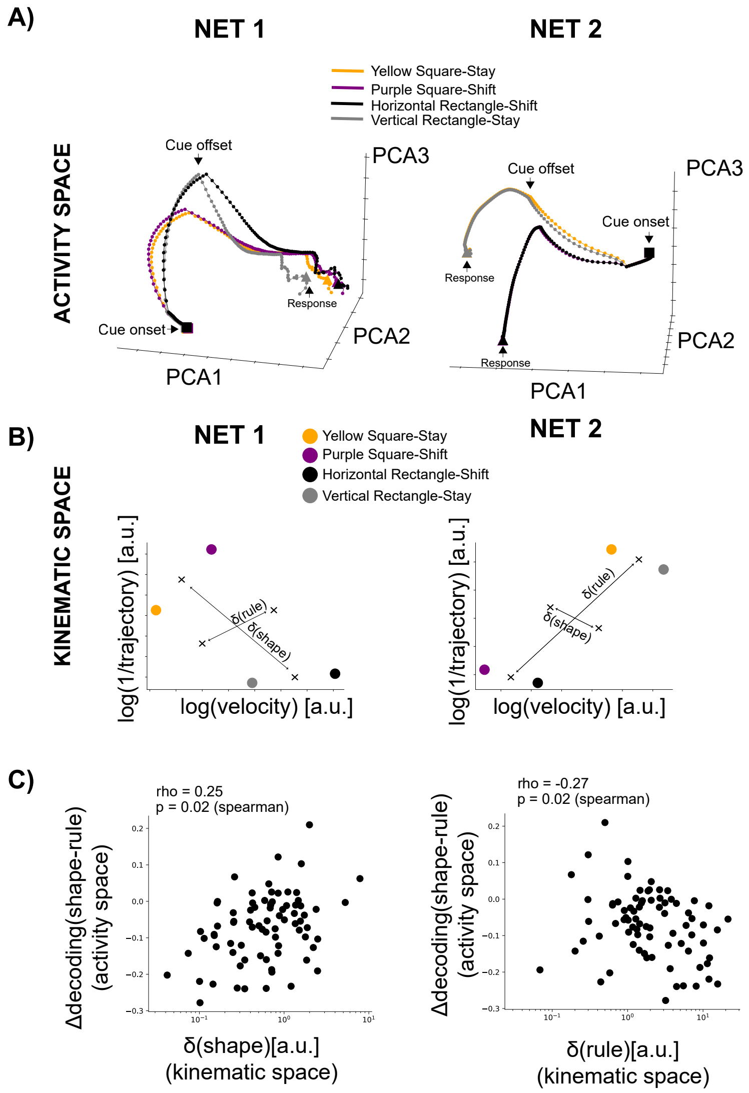

Fig. S9: **Analysis of the relation between representational geometry and reaction time in RNNs. A)** Population activity trajectory in the principal component space for the first three axes, showing the activity from cue onset to response for four visual cues for the two example neural networks (NET 1 and NET 2). These trajectories illustrate the representational geometry where, after the cue onset, trajectories with the same shapes or rules exhibit closer proximity within the activity space. **B)** The trajectory length and average velocity of the RNN population from cue onset to response define the kinematic space. Here, δ(shape) and δ(rule) are the Euclidean distances between the centroids of differing shapes and rules, respectively. **C)** Correlation, for all the RNNs, between the distance measures defined in panel B and the difference in the decoding accuracy between shape and rule.
