# Supplementary material for: Neural representational geometries reflect behavioral differences in monkeys and recurrent neural networks
Source: Nat Commun. 2024 Aug 1;15:6479. doi: 10.1038/s41467-024-50503-w (PMC11294567; doi:10.1038/s41467-024-50503-w)
Supplement: Supplementary file 3 — Reporting Summary [file 41467_2024_50503_MOESM3_ESM.pdf]

nature portfolio

Corresponding author(s): Valeria Fascianelli
Aldo Genovesio
Stefano Fusi

Last updated by author(s): 2024/06/04

Reporting Summary

Nature Portfolio wishes to improve the reproducibility of the work that we publish. This form provides structure for consistency and transparency in reporting. For further information on Nature Portfolio policies, see our Editorial Policies and the Editorial Policy Checklist.

Statistics

For all statistical analyses, confirm that the following items are present in the figure legend, table legend, main text, or Methods section.

n/a	Confirmed	
☐	☒	The exact sample size (n) for each experimental group/condition, given as a discrete number and unit of measurement
☐	☒	A statement on whether measurements were taken from distinct samples or whether the same sample was measured repeatedly
☐	☒	The statistical test(s) used AND whether they are one- or two-sided<br>*Only common tests should be described solely by name; describe more complex techniques in the Methods section.*
☐	☒	A description of all covariates tested
☒	☐	A description of any assumptions or corrections, such as tests of normality and adjustment for multiple comparisons
☐	☒	A full description of the statistical parameters including central tendency (e.g. means) or other basic estimates (e.g. regression coefficient) AND variation (e.g. standard deviation) or associated estimates of uncertainty (e.g. confidence intervals)
☐	☒	For null hypothesis testing, the test statistic (e.g. F, t, r) with confidence intervals, effect sizes, degrees of freedom and P value noted<br>*Give P values as exact values whenever suitable.*
☒	☐	For Bayesian analysis, information on the choice of priors and Markov chain Monte Carlo settings
☒	☐	For hierarchical and complex designs, identification of the appropriate level for tests and full reporting of outcomes
☒	☐	Estimates of effect sizes (e.g. Cohen's d, Pearson's r), indicating how they were calculated

Our web collection on statistics for biologists contains articles on many of the points above.

Software and code

Policy information about availability of computer code

Data collection	All of our code for this project is written in Python, making use of Pytorch (v. 1.13) and gym (v. 0.23.1) and the broader python scientific computing environment (including numpy v1.25, scipy v 1.11, matplotlib v.3.8, and scikit-learn v.1.4).
Data analysis	The codes to run all the analyses, both on neural and artificial data, and to reproduce the figures will be available at the following github repository https://github.com/ValeriaFascianelli/geometry-individual-differences.git.

For manuscripts utilizing custom algorithms or software that are central to the research but not yet described in published literature, software must be made available to editors and reviewers. We strongly encourage code deposition in a community repository (e.g. GitHub). See the Nature Portfolio guidelines for submitting code & software for further information.

Data

Policy information about availability of data

All manuscripts must include a data availability statement. This statement should provide the following information, where applicable:

- Accession codes, unique identifiers, or web links for publicly available datasets
- A description of any restrictions on data availability
- For clinical datasets or third party data, please ensure that the statement adheres to our policy

All the datasets, both neural and artificial, to reproduce the main figures are available at the following github repository https://github.com/ValeriaFascianelli/geometry-individual-differences.git

Research involving human participants, their data, or biological material

Policy information about studies with human participants or human data. See also policy information about sex, gender (identity/presentation), and sexual orientation and race, ethnicity and racism.

Reporting on sex and gender	This study does not involve any human participants
Reporting on race, ethnicity, or other socially relevant groupings	This study does not involve any human participants
Population characteristics	This study does not involve any human participants
Recruitment	This study does not involve any human participants
Ethics oversight	This study does not involve any human participants

Note that full information on the approval of the study protocol must also be provided in the manuscript.

Field-specific reporting

Please select the one below that is the best fit for your research. If you are not sure, read the appropriate sections before making your selection.

☒ Life sciences ☐ Behavioural & social sciences ☐ Ecological, evolutionary & environmental sciences

For a reference copy of the document with all sections, see nature.com/documents/nr-reporting-summary-flat.pdf

Life sciences study design

All studies must disclose on these points even when the disclosure is negative.

Sample size	No power analysis was performed before the beginning of the experiment. In non-human primates experiment, ethical and practical constraints typically limit the sample size to two animals.
Data exclusions	Error trials have been excluded from the current analyses. For neural and artificial data we analyzed only complete and correct trials. For the analyses of the reaction times, we identified as outliers data points that differed from the mean by >3 STD. Removing outliers with >3STD criterion is a standard practice in data analysis and statistics. Including outliers trials in the current study did not significantly change the results.
Replication	The experiment has been replicated across 2 subjects. The overall performance of the task has been successfully reproduced across the subjects. We trained 80 artificial networks, and we are able to reproduce the neuronal results and we show the results across all trained networks separately.
Randomization	There was not any bias in allocating any of the subjects under study to any experimental groups. All the analyses have been performed on each subject separately. All the artificial networks have been trained independently and all have different random seeds for network initialization.
Blinding	Data collection and analysis were not performed blind to the conditions of the experiments.

Reporting for specific materials, systems and methods

We require information from authors about some types of materials, experimental systems and methods used in many studies. Here, indicate whether each material, system or method listed is relevant to your study. If you are not sure if a list item applies to your research, read the appropriate section before selecting a response.

Materials & experimental systems

n/a	Involved in the study
☒	☐ Antibodies
☒	☐ Eukaryotic cell lines
☒	☐ Palaeontology and archaeology
☐	☒ Animals and other organisms
☒	☐ Clinical data
☒	☐ Dual use research of concern
☒	☐ Plants

Methods

n/a	Involved in the study
☒	☐ ChIP-seq
☒	☐ Flow cytometry
☒	☐ MRI-based neuroimaging

Animals and other research organisms

Policy information about studies involving animals; ARRIVE guidelines recommended for reporting animal research, and Sex and Gender in Research

Laboratory animals	The study involved 2 male rhesus monkeys, Macaca mulatta, 10–11kg in weight
Wild animals	The study did not involve wild animals
Reporting on sex	The study involved 2 male rhesus monkeys
Field-collected samples	The study did not involve field-collected samples
Ethics oversight	All experimental procedures were in agreement with the Guide for the Care and Use of Laboratory Animals and were approved by the National Institute of Mental Health Animal Care and Use Committee

Note that full information on the approval of the study protocol must also be provided in the manuscript.
